# Predominant Leptospiral Serogroups Circulating among Humans, Livestock and Wildlife in Katavi-Rukwa Ecosystem, Tanzania

**DOI:** 10.1371/journal.pntd.0003607

**Published:** 2015-03-25

**Authors:** Justine A. Assenga, Lucas E. Matemba, Shabani K. Muller, Ginethon G. Mhamphi, Rudovick R. Kazwala

**Affiliations:** 1 Department of Veterinary Medicine and Public Health, Sokoine University of Agriculture, Morogoro, Tanzania; 2 National Institute for Medical Research, Dar Es Salaam, Tanzania; 3 Pest Management Centre, Sokoine University of Agriculture, Morogoro, Tanzania; University of Tennessee, UNITED STATES

## Abstract

**Background:**

Leptospirosis is a worldwide zoonotic disease and a serious, under-reported public health problem, particularly in rural areas of Tanzania. In the Katavi-Rukwa ecosystem, humans, livestock and wildlife live in close proximity, which exposes them to the risk of a number of zoonotic infectious diseases, including leptospirosis.

**Methodology/Principal Findings:**

A cross-sectional epidemiological study was carried out in the Katavi region, South-west Tanzania, to determine the seroprevalence of *Leptospira spp* in humans, domestic ruminants and wildlife. Blood samples were collected from humans (n = 267), cattle (n = 1,103), goats (n = 248), buffaloes (n = 38), zebra (n = 2), lions (n = 2), rodents (n = 207) and shrews (n = 11). Decanted sera were tested using the Microscopic Agglutination Test (MAT) for antibodies against six live serogroups belonging to the *Leptospira spp*, with a cutoff point of ≥ 1:160. The prevalence of leptospiral antibodies was 29.96% in humans, 30.37% in cattle, 8.47% in goats, 28.95% in buffaloes, 20.29% in rodents and 9.09% in shrews. Additionally, one of the two samples in lions was seropositive. A significant difference in the prevalence *P*<0.05 was observed between cattle and goats. No significant difference in prevalence was observed with respect to age and sex in humans or any of the sampled animal species. The most prevalent serogroups with antibodies of *Leptospira spp* were Sejroe, Hebdomadis, Grippotyphosa, Icterohaemorrhagie and Australis, which were detected in humans, cattle, goats and buffaloes; Sejroe and Grippotyphosa, which were detected in a lion; Australis, Icterohaemorrhagie and Grippotyphosa, which were detected in rodents; and Australis, which was detected in shrews. Antibodies to serogroup Ballum were detected only in humans.

**Conclusions:**

The results of this study demonstrate that leptospiral antibodies are widely prevalent in humans, livestock and wildlife from the Katavi-Rukwa ecosystem. The disease poses a serious economic and public health threat in the study area. This epidemiological study provides information on circulating serogroups, which will be essential in designing intervention measures to reduce the risk of disease transmission.

## Author Summary

Leptospirosis is a disease of worldwide significance, and it is also an important zoonotic disease, particularly in developing countries. Subclinically infected rodents maintain leptospires in nature, and some that recover from the primary leptospiral infection may release the bacterium in their urine for the rest of their lives. These rodents serve as a potential source of leptospiral infection to animals and humans. Non-rodent mammals can also be reservoirs of leptospiral infection to animals and humans. Globally, animal and human leptospirosis has been attributed to rodents. There is limited knowledge on the occurrence of the disease in domestic animals, humans, wildlife and rodents in many parts of Tanzania, including the Katavi-Rukwa ecosystem. Serological examination of cattle, goats, humans, buffaloes, zebra, lions, rodents and shrews in the ecosystem revealed the presence of antibodies to serogroups Sejroe, Hebdomadis, Grippotyphosa, Icterohaemorrhagie, Australis and Ballum. These serogroups infect not only their usual hosts but also other animal species, which can in turn act as reservoirs of these serogroups to other animals and humans. This study demonstrates the distribution of leptospiral serogroups in domestic animals, humans, wildlife, rodents and shrews in the Katavi-Rukwa ecosystem. The results of the current study will help in developing appropriate interventions for preventing or mitigating the impacts of infections in domestic animals, humans, wildlife, rodents and shrews. Our results also suggest that human and animal populations are at risk of contracting the infection.

## Introduction

Leptospirosis is an emerging/re-emerging, worldwide, contagious, bacterial zoonotic disease that affects all mammals, including humans, livestock and wildlife [1, 2]. The disease is caused by different serovars of pathogenic species of the genus *Leptospira* [1, 2], which is common in tropical and subtropical regions, wherever environmental conditions favour the survival and transmission of the bacterium [3, 4]. Leptospirosis was first identified by Weil (1886) and Inada (1916) [5]. In the East and Central African regions, the disease was reported three decades ago [6]. The sources of infection for humans and other incidental hosts, such as cattle, pigs, horses, and companion animals, are subclinically infected wild and domestic animals, which are the reservoirs for over 250 known serovars of *Leptospira* [7]. Rodents are the most important source of infection for humans and animals [8, 9]. The role of rodents as carriers and the main source of leptospiral infection in human has been investigated in some countries. Moreover, different species of rodents, such as *Rattus*, *R*. *norvegicus*, *Mus musculus*, *Bandicota bengalensis*, *Bandicota indica* and *Cricetomys gambianus*, are known to carry different pathogenic leptospiral serovars [8, 9]. *Leptospira spp* lives for a long time in the kidney tubules of an infected animal host, from where they are excreted through the urine [10]. Humans become infected through either direct contact with the urine or other biological materials from the infected animals or indirect contact with water, soil and vegetation polluted with urine from animals harbouring pathogenic leptospires [11]. Leptospirosis is also an occupational disease affecting veterinarians, abattoir workers, sewer workers and other groups of people whose job exposes them constantly to contaminated materials [12]. A serological assay, the Microscopic Agglutination Test (MAT), is considered as the gold standard for the diagnosis of leptospiral infection [12]. The test is used to detect antibodies against different Leptospiral serovars.

Previous reports from Tanzania have indicated that leptospiral infection is widely prevalent in humans, livestock, and rodents in some parts of the country [13, 5, 14, 15, 16, 17, 18, 7]. However, a study on leptospirosis in the Katavi region has not been conducted, suggesting that the role of animals in the transmission and maintenance of the infection is not well understood. Hence, the objective of this study was to establish the seroepidemiology of *Leptospira spp* and to identify the most prevalent leptospiral serogroups in humans and animals using the Microscopic Agglutination Test (MAT).

## Materials and Methods

### Description of the study area

**Fig 1:**
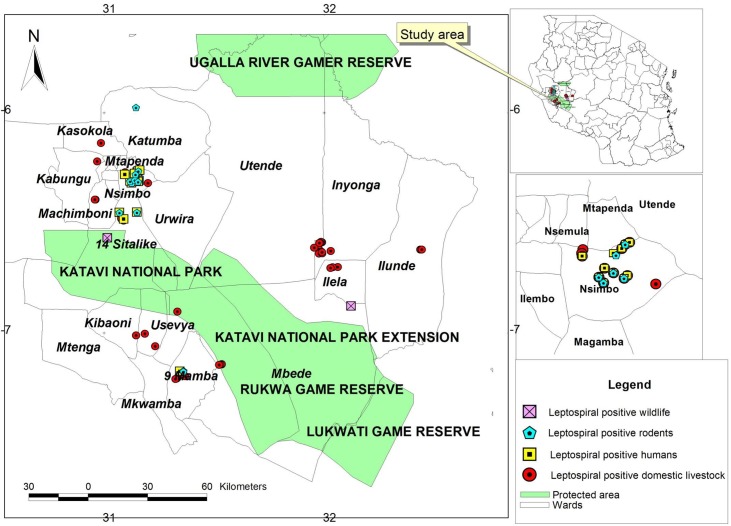
Map of the Katavi region showing the geographical distribution of leptospiral antibodies based on MAT results. The inset is a map of Tanzania showing the study area.

The study was carried out between September 2012 and April 2013 in the Katavi region, southwest Tanzania, which is an agro-pastoral community with a wide range of domestic animals and wildlife. The Katavi region is located approximately 6° 30’S and 31° 30’E. All the districts in the Katavi region, namely Mpanda, Nsimbo, and Mlele, (Fig. 1) were involved in this study. Katavi has a tropical climate, with a rainy season from November to April and a dry season from May to October. During the rainy season, rainfall can be extremely high, with a mean annual rainfall greater than 100 mm. The economic activities of the people in the Katavi region are mainly livestock keeping and small-scale farming. For livestock keeping, Katavi residents practice free-range grazing, and for small-scale farming, they cultivate both food and cash crops. During the dry season, the agro-pastoralist graze animals on crop residues, and thereafter, they shift these animals to distant grazing land, commonly known as grazing camps. Different habitats are selected for trapping rodents, such as, plough fields, tiny bushes around homes, marshy areas for the cultivation of rice and sugar, vegetable gardens, and areas with garbage close to homes and within homes.

Katavi contains national parks, such as Katavi National Park, which is composed of seasonally flooded grassland plains, miombo woodlands, small lakes, and swampy wetlands [19]. Wild animals commonly found in the park include African buffaloes (*Syncerus caffer*), elephants (*Loxondata africana*), zebras (*Equus burchelli*), impalas (*Aepyceros melampus*), giraffes (*Giraffa Camelopardalis*), elands (*Taurotragus oryx*), baboons (*Papio anubis*), hippopotamuses (*Hippopotamus amphibious*), and predators, such as lions (*Panthera leo*) and other small carnivores [20].

### Ethical consideration

The ethical clearance for conducting this study was granted by the Institutional Review Board of Sokoine University of Agriculture (SUA/FVM/R.1/9), Medical Research Coordinating Committee of the National Institute for Medical Research, reference number NIMR/HQ/R8a/Vol.IX/1627, and the Tanzania Wildlife Research Institute (TAWIRI). Additionally, permission was requested and granted from all local authorities in the study area, including TANAPA and the Local Government Authority. Verbal consents were obtained from all the study participants. To safeguard the wellbeing of animals, this study adhered to Animal Welfare Act [21], as well as the guidelines adopted from the Australian government [22].

### Study design and study subjects

A cross-sectional epidemiological study was carried out, in which a multistage cluster sampling was conducted. Villages were randomly selected as the primary unit, from which a total of 138 households were chosen from the list of agro-pastoralists using random numbers. Members of the selected households were subjected to a random selection to obtain a total of 267 humans who were readily available, regardless of their health status. Our target was households with domestic animals. Thus, using cluster sampling, a total of 1351 apparently healthly livestock were selected from the same households where humans were sampled, as described above [23]. Calves and kids below three months and children below the age of two years were not sampled. Wild animals were also targeted for this study, and a total of 42 of these animals were sampled opportunistically. Rodents and shrews were trapped from the sites located near human settlements and near human activities, including homes and crop stores in open fields where a large number of rodent burrows were observed. A total of 207 live rodents and 11 shrews were captured using Sherman LFA Live traps (7.5 × 9.0 × 23.0 cm; HB Sherman Traps, Inc., Tallahassee, FL). The traps were placed inside and surrounding the selected houses early in the evenings, and peanut butter mixed with concentrates was used as bait. The traps were inspected every morning, and the captured rodents were anaesthetized using ether in cotton swabs before taking samples. The sex, species, weight, age, and location of the trappings of the captured rodents and shrews were identified and recorded. The rodents and shrews were grouped into two classes based on age (juvenile and adult), as previously described [24].

### Sample collection and handling

Human blood samples were collected from the brachial vein using 5 ml plain vacutainer tubes. Cattle and goats were manually restrained, and blood samples were collected from the jugular vein using 10 ml plain vacutainer tubes. For wild animals, buffaloes were captured by darting using a combination of 5–8 mg etorphine hydrochloride (M99 9.8 mg/ml) (Novartis, Kempton Park, South Africa) and 50–80 mg azaperone tartarate, and zebras were immobilized using a combination of 6–7 mg etorphine hydrochloride (M99) and 80 mg azaperone. Lions were immobilized using a combination of 2.5 mg/kg ketamine hydrochloride and 0.1 mg/kg medetomidine hydrochloride (Kyron, Pty, SA). The drug was remotely injected using a darting gun. The antidote, diprenorphine hydrochloride (M5050) (Novartis, Kempton Park, South Africa), was used to revive the buffaloes and zebras after the collection of the blood samples. Lions were revived using antisedan (atipemazole hydrochloride). The blood from these animals was collected from the jugular vein using 10 ml plain vacutainer tubes. The blood samples from both domestic and wild animals were allowed to clot in a slanted position, and serum samples were harvested after 24 hours.

For rodents and shrews, blood was collected from the retro orbital sinus using sterile capillary tubes and then transferred to eppendorf tubes. The samples were centrifuged, and sera were immediately harvested.

The sera harvested from domestic animals, wildlife, rodents and shrews were dispensed into appropriately labelled 1.5 ml cryovials and stored in liquid nitrogen (−78°C) before being transferred to the Faculty of Veterinary Medicine, Sokoine University of Agriculture laboratories and stored in an ultra-deep freezer (−80°C) until a subsequent MAT was performed.

### Microscopic Agglutination Test for detection of leptospiral antibodies

Seven leptospiral serogroups, including local isolates, Icterohaemorrhagie (*Leptospira interrogans* serovar Sokoine), Australis (*Leptospira interrogans* serovar Lora), Ballum (*Leptospira borgpetersenii* serovar Kenya) and Grippotyphosa (*Leptospira kirschneri* serovar Grippotyphosa), and reference serogroups, Sejroe (*Leptospira interrogans* serovar Hardjo), Hebdomadis (*Leptospira santarosai* serovar Hebdomadis) and Canicola (*Leptospira interrogans* serovar Canicola), which are commonly found in Tanzania, were used in the study.

All sera were tested for antibodies against live antigens suspensions of *Leptospira spp* serogroups Icterohaemorrhagie (Sokoine), Australis (Lora), Ballum (Kenya), Gripotyphosa (Grippotyphosa), Sejroe (Hardjo), Hebdomadis (Hebdomadis) and Canicola (Canicola) by MAT, as previously described by [25] and [26]. Briefly, the sera (10 μl) were diluted with phosphate buffered saline (PBS) to obtain 100 μl of diluted sera in ‘U’ microtitration plates to obtain an initial dilution range of 1:20–1:160. Then, 50 μl of the full-grown antigens in Ellighausen—mcCoullough/Johnson-Harris (EMJH) with an approximate density of 3*10^8^ leptospires/ml on the MacFarland scale was added to all microtiter plate wells and mixed thoroughly on a microshaker. The microtitration plates were then incubated at 30°C for two hours. The serum antigen mixture was visualized under dark field microscopy for the presence of agglutination/clearance, and the titers were then determined. A serum was considered positive if 50% or more of the microorganisms in the microtiter well were agglutinated at the titer ≥ 1: 160. This was determined by comparing 50% of spirochaetes, which remained free cells with a control culture diluted 1:2 in phosphate-buffered saline [26]. In this study, we examined the positive and negative controls and selected the samples that agglutinated more than halfway through, as previously described by International Committee on Systematic Bacteriology [27]. The samples that agglutinated were identified during the screening of 1:160 dilutions, the numbers were recorded, and the sera were further diluted to determine the end point titer for each sample. The agglutinating sera were tested again at dilutions of 1:20, 1:40, 1:80, 1:160, 1:320, 1:640, 1:1,280, 1:2,560, 1:5,120, 1: 10,240 and 1:20,480. Negative and positive controls were included in each test. Phosphate-buffered saline (PBS) was used as a negative control. As a negative control, an equal (50 μl) volume of PBS was mixed with the different antigens. The positive control used in this study was rabbit antiserum of each specific serogroup. The positive control antiserum was supplied by the WHO Reference laboratory at the Royal Institute of Hygiene (KIT), Amsterdam, Netherlands. We used seven different positive control antisera from rabbit to test samples from animals and humans, regardless of the species tested.

### Data analysis

Microsoft Office Excel^®^ 2007 (Microsoft Corporation, Redmond, 98052-7329, USA) was used for storing data and drawing graphs. The prevalence of leptospiral antibodies was computed using Epi-Info version 7 (CDC Atlanta, USA). The proportions were compared using MedCalc^®^ version 13.0.2 (MedCalc software, Acacialaan 22, B-8400, Ostend, Belgium).

## Results

### Leptospira seroprevalence

**Fig 2:**
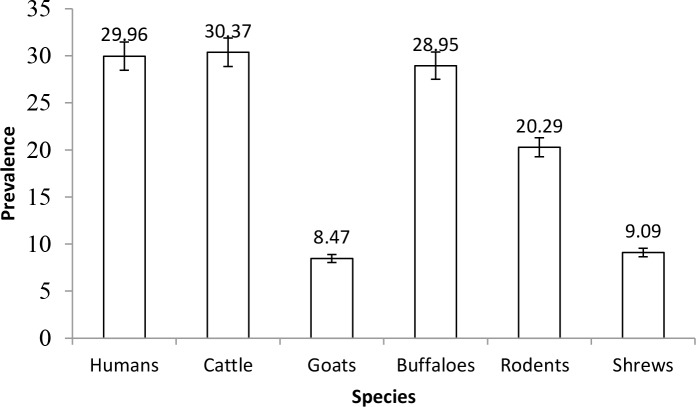
Prevalence of leptospiral antibodies in humans, cattle, goats, buffaloes, rodents and shrews in the Katavi region, Tanzania.

The overall prevalences of leptospiral antibodies in human, domestic ruminants, wildlife, rodents and shrews were 29.96%, 26.35%, 28.57%, 20.29% and 9.09%, respectively. The specific prevalences of leptospiral antibodies in human and in different animal species are indicated in Fig. 2. One of the two sampled lions was seropositive. Statistical analysis of the results for cattle and goats demonstrated a significant difference in seroprevalence between the two species (difference 21.90%, 95% CI = 16.93–26.12, *P*<0.0001). No leptospiral antibodies were detected in zebra (n = 2). The association of leptospiral infection with sex and age in humans, cattle, goats, rodents, and shrews was not statistically significant (*P* > 0.05).

In this study, leptospira antibodies were detected in 42 (20.29%) out of 207 apparently healthy rodents tested. Additionally, 11 shrews were tested, and one (9.09%) was found positive. One shrew and seven rodent species were found positive, with varying prevalence among species. The prevalences of leptospira antibodies among different rodent and shrew species are shown in Table 1. The geographical distribution of leptospiral antibodies were based on the MAT results and are shown in Fig. 1.

**Table 1:** Prevalence of leptospiral antibodies in different species of rodents and shrews in the Katavi region, Tanzania.

Species	Number captured and tested	No positive	%positive
**Rodents**			
*Aesthomys chrysophilus*	8	3	37.50
*Dasmys incomtus*	4	1	25
*Mastomys natalensis*	90	19	21.11
*Rattus rattus*	2	1	50
*Lemniscomys griselda*	4	2	50
*Lemniscomys rosalia*	6	0	0
*Gerbilliscus vicinus*	93	16	17.24
**Total**	**207**	**42**	**20.29**
**Shrews (Insectivores)**			
*Crocidura hirta*	11	1	9.09
**Total**	**11**	**1**	**9.09**

**Fig 3:**
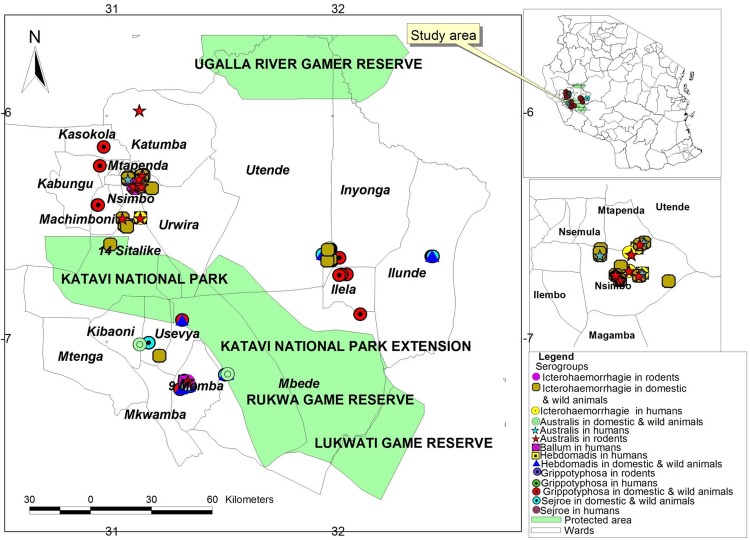
Map of the Katavi region showing the distribution of leptospiral serogroups in humans, domestic and wild animals and rodents. The inset is a map of Tanzania showing the study area.

The proportions of seropositive individuals exposed to different serogroups, for humans, domestic ruminants and rodents, are presented in Table 2. Australis was the only serogroup exposed to shrews. In buffaloes, the detected antibodies specific for the serogroups were Sejroe (7.89%), Hebdomadis (7.89%), Australis (5.26%), Grippotyphosa (5.26%), and Icterohaemorrhagie (5.26%), and antibodies against Sejroe and Grippotyphosa were detected in one of the two lions. The results also showed that samples from 13 humans, 63 cattle, two goats, and two rodents reacted to more than one serogroups (Table 3). In buffaloes, the three positive samples showed serological cross-reactions with two serogroups, specifically, Icterohaemorrhagie and Sejroe, Australis and Grippotyphosa and Hebdomadis and Icterohaemorrhagie. With regards to the lion, the positive sample showed cross-reactions of serogroups Sejroe and Grippotyphosa. The distributions of the different serogroups among the seropositive humans, domestic ruminants, wildlife, rodents and shrews are shown in Fig. 3.

**Table 2:** Seroprevalence of different leptospiral serogroups in cattle, goats, humans and rodents in the Katavi region, Tanzania.

Serogroups	Cattle		Goats		Humans		Rodents	
	No positive	%	No positive	%	No positive	%	No positive	%
Hebdomadis	85	7.71	2	0.81	9	3.37	0	0
Australis	9	0.82	1	0.40	4	1.49	39	18.84
Ballum	0	0	0	0	3	1.12	0	0
Grippotyphosa	53	4.81	4	1.61	13	4.87	1	0.48
Icterohaemorrhagie	52	4.71	8	3.23	24	8.99	4	1.93
Sejroe (Hardjo)	194	17.59	7	2.82	42	15.73	0	0

**Table 3:** Co-agglutination of different leptospiral serogroups in cattle, goats, humans and rodents in the Katavi region, Tanzania.

Serogroups	Cattle		Goats		Humans		Rodents	
	No positive	%	No positive	%	No positive	%	No positive	%
Sejroe and Grippotyphosa	13	1.18	0	0	2	0.75	0	0
Sejroe and Icterohaemorrhagie	3	0.27	0	0	3	1.12	0	0
Sejroe and Australis	2	0.18	0	0	0	0	0	0
Sejroe and Hebdomadis	20	1.81	0	0	1	0.37	0	0
Hebdomadis and Icterohaemorrhagie	8	0.73	1	0.40	2	0.75	0	0
Hebdomadis and Grippotyphosa	2	0.18	0	0	0	0	0	0
Australis and Grippotyphosa	1	0.09	0	0	0	0	1	0.46
Ballum and Sejroe	0	0	0	0	1	0.37	0	0
Icterohaemorrhagie and Grippotyphosa	0	0	0	0	3	1.12	0	0
Australis and Icterohaemorrhagie	0	0	0	0	0	0	1	0.46
Icterohaemorrhagie, Hebdomadis and Sejroe	1	0.09	0	0	0	0	0	0
Hebdomadis, Grippotyphosa and Sejroe	4	0.36	0	0	0	0	0	0
Hebdomadis, Australis and Sejroe	1	0.09	0	0	0	0	0	0
Icterohaemorrhagie, Grippotyphosa and Sejroe	0	0	0	0	1	0.37	0	0

## Discussion

The findings from this study indicate that leptospiral antibodies are prevalent in the Katavi-Rukwa ecosystem, as the antibodies were detected in humans, cattle, goats, buffaloes, lions, rodents and shrews. This is the first report of leptospira seroprevalence linking humans and animal infections in Tanzania. The demonstration of the exposure of these animals and humans at the same time provides a significant and important epidemiological picture and increases our understanding of infection patterns of leptospiral serogroups at the interface areas. Previous studies demonstrated that the seroprevalence of leptospira in healthy animals suggests levels of local exposure [28]. Animals with low prevalence of leptospira antibodies might be a significant cause of infection in humans, and high seroprevalence may signify exposure pressure from different animals and thus a high infection risk in humans as well [28].

In domestic animals, the highest seroprevalence was observed in cattle, as opposed to goats, and the difference was statistically significant (*P<*0.0001). The observed difference can be attributed to the feeding behaviour of goats, specifically, grazing on the top end of grasses, browsing on shrubs and staying in less wet areas, as opposed to cattle. As such, they have less exposure to leptospires [29]. In the present study, no significant difference in seroprevalence according to age was established in humans and in other animal species. The study results demonstrate that leptospirosis is endemic in the study area. This implies that all age groups face equal risk of being infected by leptospires. This finding is in agreement with the observation made by other researchers [30].

In humans, the serogroup Sejroe (serovar Hardjo) was the predominant serogroup, followed by Icterohaemorrhagie. Other prevalent serogroups were Grippotyphosa, Hebdomadis, Ballum and Australis. The predominance of serogroup Sejroe (serovar Hardjo) in humans can be attributed to the high contact rate with cattle, which are widespread in areas where human subjects were sampled for this serosurvey. Cattle are known to be natural hosts for serovar Hardjo, and the spirochete can survive in cattle for years [31]. Interactions between humans and cattle can lead to the interspecies transmission of serovar Hardjo. The seropositivity of serogroup Icterohaemorrhagie (serovar Sokoine) and Grippotyphosa in humans can be attributed to the abundance of rodents in the study area, as rodents are the natural carriers of these serogroups [31].

Antibodies to serogroup Ballum (serovar Kenya) were detected in humans but not in the sampled animal species in the ecosystem. The serogroup was previously isolated from urine of African giant pouched rat (*Cricetomys gambianus*) from Morogoro, Tanzania [14]. The seropositivity of the serogroup Ballum in humans may be due to the presence of African giant pouched rats in the study area, which may serve as a potential source of the serogroup to humans due to contamination of the environment with urine.

The main serogroup identified in cattle was Sejroe (serovar Hardjo). This finding corresponds well with findings in previous published reports that showed that cattle are the maintenance host of this serogroup [5, 18]. However, studies conducted in different areas of Tanzania have reported significant lower seroprevalence (5.6%) than what was observed in the current study [5]. The observed difference in the results between the current and the previous studies is likely due to variations in ecological factors, such as humidity, climate, and environmental factors [32, 33], as well as a variation in the level of interaction with other animals in the study area. Serovar hardjo is considered to be an important cause of bovine leptospirosis, which, in most cases, has been associated with abortion in cattle and has also been the most common cause of leptospiral infection in humans, due to the possibility of high rates of interaction between cattle and humans [31]. Other serogroups detected in cattle, such as Hebdomadis, Australis, Grippotyphosa and Icterohaemorrhagie, are accidental infections that are carried by other domestic and free range animals, and which are dependent on farm management practices, as described elsewhere [34].

Icterohaemorrhagie, Sejroe (serovar Hardjo) and Grippotyphosa were the most prevalent serogroups observed in goats. Similarly, rodents are known to be the natural reservoir hosts for the serogroups, Icterohaemorrhagie and Grippotyphosa [31]. Therefore, the high prevalence of these two serogroups in goats implies that there is probably high rate of interaction between goats and rodents in the study area. Furthermore, the results suggest interaction between these animals and humans, as the same serogroups were detected in human samples in the same interface (Table 2). The serogroups Icterohaemorrhagie (serovar Sokoine) and Sejroe (serovar Hardjo) have previously been reported to be among the most important occupational diseases in and around Tanga city, eastern Tanzania [35].

Antibodies to different leptospiral serogroups were detected in seven different species of rodents (Table 1) trapped in various areas of the Katavi-Rukwa ecosystem, suggesting that rodents are probably the carriers of different leptospiral serogroups, therefore exacerbating transmission of leptospiral infection to humans and animals in the ecosystem. Serogroup Australis had the highest seroprevalence (18.84%), followed by Icterohaemorrhagie (1.93%) and Grippotyphosa (0.48%), in the tested rodents in the study area. These findings are in agreement with the findings in previous studies conducted in different parts of Tanzania [11, 7]. Interactions among rodents, humans, domestic ruminants and wildlife occur frequently in the study area, as rodents share the same habitat with these animals and humans. These interactive activities of rodents in the study area create a favourable environment for leptospiral transmission from rodents to humans, domestic animals and wildlife. Australis was the only serogroup exposed to shrews, with a seroprevalence of 9.09%. Exposure to the serogroup was also detected in both rodents and shrews (insectivores). This study was not able to demonstrate whether shrews were the maintenance host for this serogroup or if the serogroup was transmitted to shrews from rodents. This lack of clear understanding of the maintenance hosts may require further studies in this area, as it poses a major public health risk.

In buffaloes, the predominant serogroups were Sejroe (serovar Hardjo), Hebdomadis, Australis, Grippotyphosa and Icterohaemorrhagie. The seropositivity against serogroup Serjoe (serovar Hardjo) as a predominant serogroup is probably due to high interaction with cattle, which are the maintenance host of the serogroup [36]. Buffaloes are also a reservoir for Hardjo. Hence, the possibility of transmission of the serovar from cattle to the buffaloes and vice versa is very high. The prevalence of serogroup Grippotyphosa and Icterohaemorrhagie in buffaloes may be attributed to the high rate of interaction between buffaloes and rodents in the area because rodents are the carriers of the serogroups [33]. As noted earlier, the leptospiral serogroups found in the sampled buffaloes were similar to the serogroups detected in cattle. The presence of a wide range of buffaloes in Katavi allows cattle and buffaloes to share grazing grounds and watering points. Hence, the possibility of transmission or spillover of serogroups from cattle to buffaloes and vice versa is very high. A similar observation was also reported in Turkey, where researchers found a similar leptospirosis seroprevalence in buffaloes as that observed in cattle [36]. In this study, Sejroe (serovar Hardjo) and Grippotyphosa were the only leptospira serogroups detected in lions (only two lions were sampled and only one was seropositive). These same serogroups were also found in buffaloes and domestic ruminants. This may be attributed to the feeding behaviour of lions that prey on wild and domestic ruminants.

The current study identified similar leptospiral serogroups circulating in humans, domestic ruminants, wildlife, rodents and shrews sharing the same ecosystem (Fig. 3). This may be attributed to the intense overlap of these species, bush meat handling, and environmental and seasonal drivers, such as drought and floods [37]. Katavi residents are mainly agro-pastoralists, who frequently come into contact with livestock, as well as wildlife and their excreta, in the ecosystem. Furthermore, the majority of the communities in the study area slaughter animals at home, and some of the people consume raw kidney and liver, as was reported in the interviews conducted. It is believed that direct contact between humans and animals is an important risk factor for human Leptospirosis [38, 5, 39, 40]. The results of this study indicate that livestock share the same serogroup with humans, and this implies a public health risk, particularly among those involved in animal handling. Similar findings were observed in Italy, where patients were infected through direct contact with infected animals or through contaminated urine [41].

In Katavi National Park, wild ungulates are found in high densities around lake Chada, the Kitusunga flood plains, and around lake Katavi, especially during the dry season, due to an influx of wildlife in search of pasture and water [20]. The large influx of animals might easily contaminate the area with urine and increase the chances of the spillover of infections to other animal species. During the rainy season, rivers flood, which increases the risks of leptospirosis outbreaks due to runoff soil contaminated with urine from domestic animals, wildlife, rodents and shrews flowing into common water sources. This is an important driver of leptospiral transmission.

In the Katavi-Rukwa ecosystem, bush meat consumption is common, and the main species hunted are impala, common duiker, warthog, buffalo and bushbuck [19]. Leptospiral infection in humans can occur through the direct contact with the blood, tissues, organs and urine of infected animals [39, 40]. Therefore, slaughtering and handling of bush meat from infected animals may pose a great risk of leptospiral transmission to humans in areas where consumption of bush meat is practiced. A study carried out in South America showed that human leptospirosis was associated with men who captured, slaughtered, and consumed large rodents [42].

This study observed serum agglutination to more than one serogroup in humans and all animal species tested. This may reflect a mixed or two different past infections, which most frequently reflect serological cross-reactions. These cross-reactions are mostly seen in acute or early convalescent sera, whereby the host, previously infected with one serogroup, may subsequently become infected by another serogroup, and the recently acquired serogroup may cross-react to the previous one, leading to activation of the memory response against the subsequent serogroup [43]. The titer of antibodies relating to the previous serogroup could be higher than antibodies specific to the new infecting serogroup. This may also reflect an infection caused by a serogroup not included in the MAT panel, as the MAT panel used was not very wide.

In conclusion, the present study demonstrates the possible interaction between livestock, wildlife, and humans, as similar serogroups were detected among these species. This may have a very serious implication on the public health of the communities, as the capacity to diagnose leptospirosis is not available in any of the surveyed villages, including the district hospital. Therefore, human leptospirosis should be included in the differential diagnosis of febrile illnesses in humans in the study area. Furthermore, the results from this study demonstrate common serogroups circulating among humans, domestic ruminants and wildlife, which will help in planning for interventions for the control or mitigation of the impact of infections in domestic animals and in humans. Thus, we recommend further studies on the molecular typing of leptospiral isolates from humans and from different animal species in the Katavi- Rukwa ecosystem.

## Supporting Information

S1 Text STROBE checklist. (DOC)

S1 Table Leptospira strains (antigens) used in this study. (DOC)
