# Supplementary material for: Predominant Leptospiral Serogroups Circulating among Humans, Livestock and Wildlife in Katavi-Rukwa Ecosystem, Tanzania
Source: PLoS Negl Trop Dis. 2015 Mar 25;9(3):e0003607. doi: 10.1371/journal.pntd.0003607 (PMC4373666; doi:10.1371/journal.pntd.0003607)
Supplement: S1 Text — (DOC) [file pntd.0003607.s001.doc]

**S1 Text : STROBE CHECKLIST**

STROBE Statement—Checklist of items that should be included in reports of ***cross-sectional studies***

|  | Item No | Recommendation |
| --- | --- | --- |
| **Title and abstract** | 1 | (*a*) Indicate the study’s design with a commonly used term in the title or the abstract  Yes: pages 1-2 |
|  |  | (*b*) Provide in the abstract an informative and balanced summary of what was done and what was found  Yes: pages 1-2 |
| Introduction | | |
| Background/rationale | 2 | Explain the scientific background and rationale for the investigation being reported  Yes: pages 3-5 |
| Objectives | 3 | State specific objectives, including any prespecified hypotheses  Yes: pages 4-5 |
| Methods | | |
| Study design | 4 | Present key elements of study design early in the paper  Yes: pages 6-7 |
| Setting | 5 | Describe the setting, locations, and relevant dates, including periods of recruitment, exposure, follow-up, and data collection  Yes: pages 5-8 |
| Participants | 6 | (*a*) Give the eligibility criteria, and the sources and methods of selection of participants  Yes: pages 6-7 |
| Variables | 7 | Clearly define all outcomes, exposures, predictors, potential confounders, and effect modifiers. Give diagnostic criteria, if applicable  Yes: pages 8-9 |
| Data sources/ measurement | 8* | For each variable of interest, give sources of data and details of methods of assessment (measurement). Describe comparability of assessment methods if there is more than one group  Yes : pages 6-8 |
| Bias | 9 | Describe any efforts to address potential sources of bias  Yes: page 6 |
| Study size | 10 | Explain how the study size was arrived at  Yes: page 6 |
| Quantitative variables | 11 | Explain how quantitative variables were handled in the analyses. If applicable, describe which groupings were chosen and why  Yes: page 10 |
| Statistical methods | 12 | (*a*) Describe all statistical methods, including those used to control for confounding  Yes: page 10 |
|  |  | (*b*) Describe any methods used to examine subgroups and interactions  Yes: page 10 |
|  |  | (*c*) Explain how missing data were addressed  NA |
|  |  | (*d*) If applicable, describe analytical methods taking account of sampling strategy  Yes: page 10 |
|  |  | (*e*) Describe any sensitivity analyses  NA |
| Results | | |
| Participants | 13* | (a) Report numbers of individuals at each stage of study—eg numbers potentially eligible, examined for eligibility, confirmed eligible, included in the study, completing follow-up, and analysed  Yes: pages 1,6, 7 |
|  |  | (b) Give reasons for non-participation at each stage  NA |
|  |  | (c) Consider use of a flow diagram  NA |
| Descriptive data | 14* | (a) Give characteristics of study participants (eg demographic, clinical, social) and information on exposures and potential confounders  Yes: page 10 |
|  |  | (b) Indicate number of participants with missing data for each variable of interest  NA |
| Outcome data | 15* | Report numbers of outcome events or summary measures  Yes: pages 10-11 |
| Main results | 16 | (*a*) Give unadjusted estimates and, if applicable, confounder-adjusted estimates and their precision (eg, 95% confidence interval). Make clear which confounders were adjusted for and why they were included  Yes: page 10 |
|  |  | (*b*) Report category boundaries when continuous variables were categorized  NA |
|  |  | (*c*) If relevant, consider translating estimates of relative risk into absolute risk for a meaningful time period  NA |
| Other analyses | 17 | Report other analyses done—eg analyses of subgroups and interactions, and sensitivity analyses  NA |
| Discussion | | |
| Key results | 18 | Summarise key results with reference to study objectives  Yes: pages 11-17 |
| Limitations | 19 | Discuss limitations of the study, taking into account sources of potential bias or imprecision. Discuss both direction and magnitude of any potential bias  NA |
| Interpretation | 20 | Give a cautious overall interpretation of results considering objectives, limitations, multiplicity of analyses, results from similar studies, and other relevant evidence  Yes: pages 11-17 |
| Generalisability | 21 | Discuss the generalisability (external validity) of the study results  Yes: page 17 |
| Other information | | |
| Funding | 22 | Give the source of funding and the role of the funders for the present study and, if applicable, for the original study on which the present article is based  Yes |

*Give information separately for exposed and unexposed groups.

**Note:** An Explanation and Elaboration article discusses each checklist item and gives methodological background and published examples of transparent reporting. The STROBE checklist is best used in conjunction with this article (freely available on the Web sites of PLoS Medicine at http://www.plosmedicine.org/, Annals of Internal Medicine at http://www.annals.org/, and Epidemiology at http://www.epidem.com/). Information on the STROBE Initiative is available at www.strobe-statement.org.
