# Supplementary material for: Predominant Leptospiral Serogroups Circulating among Humans, Livestock and Wildlife in Katavi-Rukwa Ecosystem, Tanzania
Source: PLoS Negl Trop Dis. 2015 Mar 25;9(3):e0003607. doi: 10.1371/journal.pntd.0003607 (PMC4373666; doi:10.1371/journal.pntd.0003607)
Supplement: S1 Table — (DOC) [file pntd.0003607.s002.doc]

**S2 Table: LEPTOSPIRA STRAINS (ANTIGENS) USED IN THIS STUDY.**

| **Serogroup** | **Serovar** | **Species** | **Reference** |
| --- | --- | --- | --- |
| Icterohaemorrhagie | Sokoine | *Leptospira kirschneri* | Mgode *et al*., 2006 |
| Australis | Lora | *Leptospira interrogans* | Faine *et al*., 1999 |
| Ballum | Kenya | *Leptospira borgpetersenii* | Machang’u *et al*., 2004 |
| Grippotyphosa | Grippotyphosa | *Leptospira kirschneri* | Faine *et al.,* 1999 |
| Sejroe | Hardjo | *Leptospira interrogans* | Faine *et al*., 1999 |
| Hebdomadis | Hebdomadis | *Leptospira santarosai* | Faine *et al*., 1999 |
| Canicola | Canicola | *Leptospira interrogans* | Faine *et al.*, 1999 |
